# Effect of biological shells aggregate on the mechanical properties and sustainability of concrete

**DOI:** 10.1038/s41598-024-61301-1

**Published:** 2024-05-09

**Authors:** Xianpeng Wang, Haoxuan Yu, Fulong Li, Kovshar Sergey Nikolayevich, Haojue Yu, Leonovich Sergey Nikolaevich, Wenbing Fan

**Affiliations:** 1 Department of Building Materials and Construction Technology, Faculty of Civil Engineering, Belarusian National Technical University, 220013 Minsk, Belarus; 2 Hainan College of Vocation and Technique, No.95 Nanhai Avenue, Longhua District, Haikou, 570105 Hainan China; 3 School of Energy and Mines, China University of Mining and Technology (Beijing), Beijing, 100083 China

**Keywords:** Aggregate concrete, Shell aggregate, Mechanical properties, Carbon emission, Economic analysis, Ecology, Ecology, Environmental sciences, Engineering, Materials science

## Abstract

The recycling bio-waste shells problem has grown more and more serious in recent years and many efforts have been made to solve this problem. One possible solution is to put these bio-shells into concrete and recycle them as building materials using the aggregate matrix concrete approach. To verify the engineering feasibility, the mechanical properties of bio-shells aggregated concrete were invested via gradient substitution rates at 10%, 30%, and 50% with a total of 78 groups of specimens in this paper. Our results show that the mechanical properties of the concrete were enhanced in maximum flexural strength and maximum compressive. Economic performance was also analyzed and found that the costs of frame-shear structure, frame structure, and tube-in-tube structure were reduced by 10.2%, 10%, and 10.3%. The carbon environmental assessment also shows superiority in the carbon reduction of a single specimen with various rates of the shell. In summary, compared with ordinary concrete materials, it is very possible to use waste bio-shells as a substitute for aggregates to develop the sustainable recycling development of concrete materials.

## Introduction

**Figure 1:**
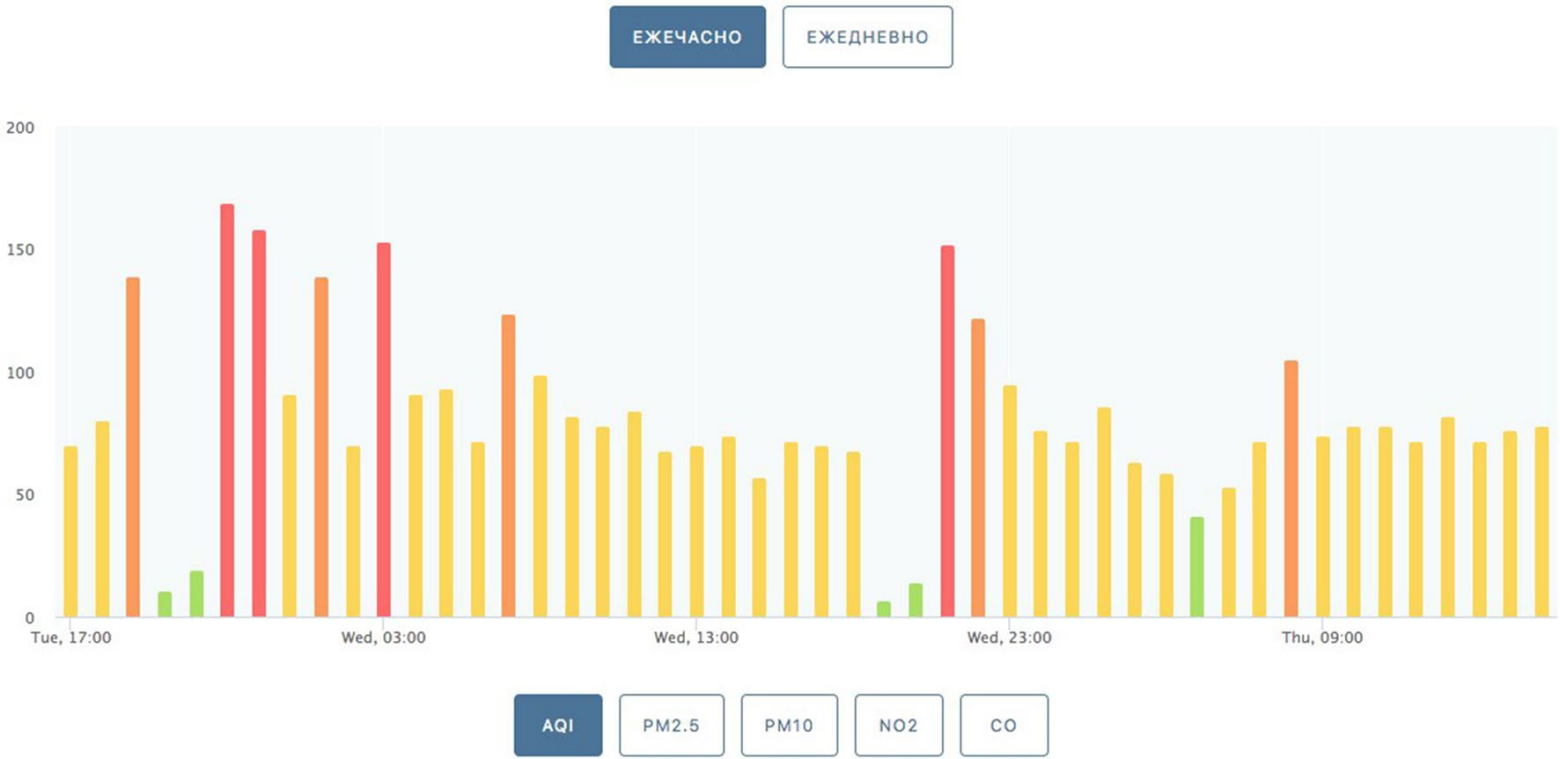
Air quality indicators^1^.

**Figure 2:**
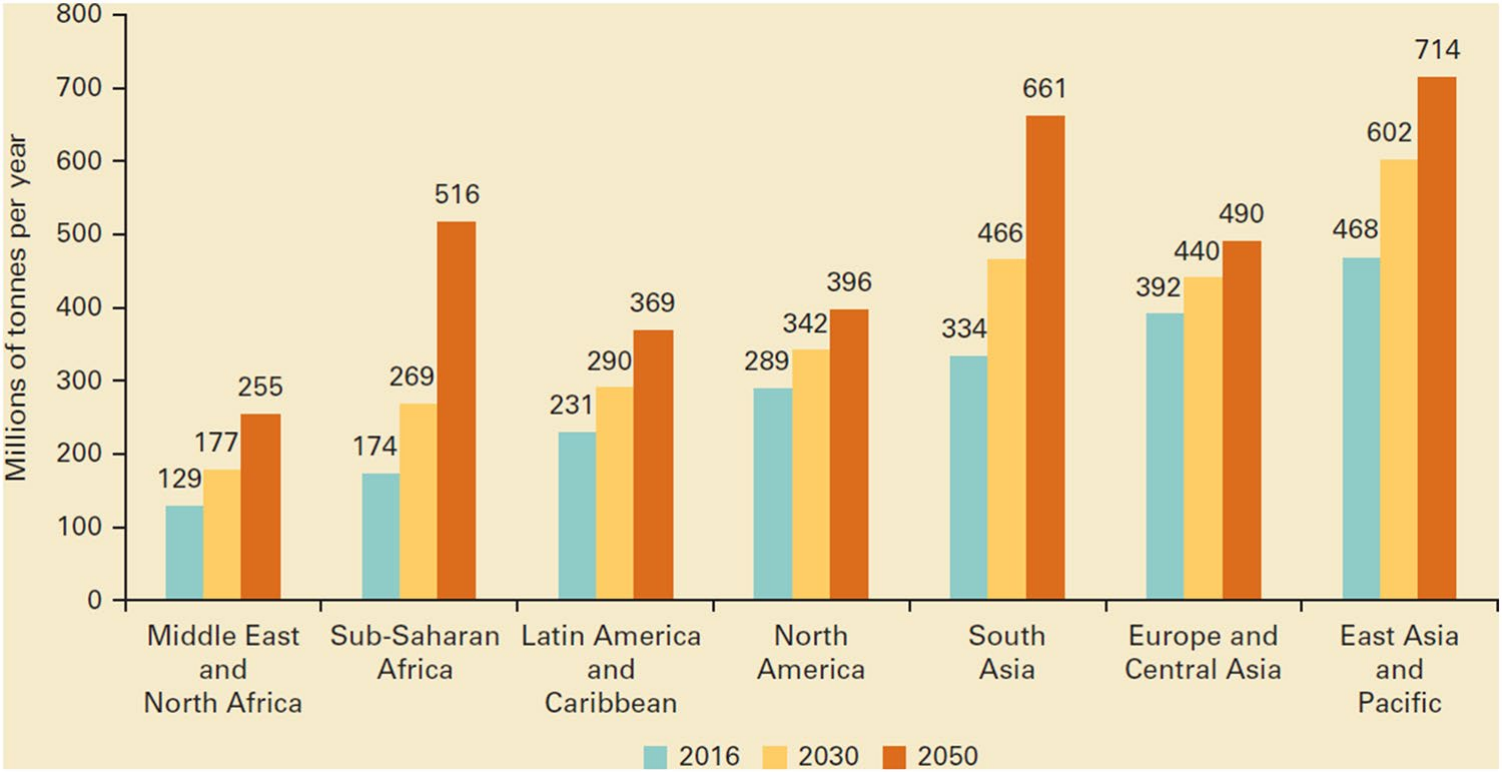
Global waste generation.

In recent years, the treatment of garbage problems has become serious all over the world, large amounts of garbage have caused serious air pollution and ecological damage, as shown in Figs. 1 and 2. To solve this problem, separating useful material from waste and using it for developing new building materials is considered a clean and economical approach to solving the waste problem. Therefore, relevant classification and composition screenings of the collected waste are done in this paper and show that bio-shells, one of the kitchen waste, can be good choices for replacing traditional concrete aggregates. As reported by previous researchers, nearly 10 million metric tons of shells of oysters, clams, scallops, and mussels are discarded globally every year^2^. Furthermore, a comprehensive survey was carried out to assess the situation of restaurants in selected provinces of China and the Republic of Belarus, the collected data revealed that the Belarusian restaurant chain brand “Sea Food” alone generates amounting to over 100 catties of discarded seashells per day. Correspondingly, approximately 5 tons of seashells are discarded daily in the provinces of Guangdong and Fujian, China^3^.

To address the pollution problem, analyzing the chemical composition and potential use of shells as building materials is necessary. The literature results have shown that shells contain calcium carbonate, glycoprotein, proteoglycan, polysaccharide, and chitin. In the hydration process of cement, water reacts with four main clinker minerals. At room temperature, tricalcium silicate hydrates form calcium silicate hydrate (C–S–H gel) and calcium hydroxide, while dicalcium silicate (β-C2S) hydrates produce similar products. Tricalcium aluminate hydrates to form unstable calcium aluminate hydrate, which eventually transforms into hydrogarnet (C3AH6). The hydration reaction of iron phase solid solution (C4AF) is like that of tricalcium aluminate^4^. Observing this hydration reaction can lead to the conclusion that shells do not affect the hydration process of cement and thus do not cause damage to the structure and strength of concrete. Therefore, shells can be used as part of concrete aggregates to develop new building materials as a recycling solution.

In coastal regions, the primary source of construction sand is desalinated sea sand, containing typically a shell content of 5–8%. From research on mortar, the incorporation of shells in place of river sand has a notable influence on the overall compressive strength and dry shrinkage of cement-based materials, with an increase of 8% in the proportion of shells^5^. Yang et al., have focused on studying shell aggregates as an alternative to natural aggregates. They suggest that shell aggregates hold promising potential for practical applications, offering a favorable solution to the growing shortage of natural aggregate resources. Research from MO’s group asserts that there is a growing inclination towards sustainability in the field of concrete engineering, primarily driven by the depletion of materials traditionally employed in the production of natural concrete. It is suggested that utilizing shell waste in the production of concrete can enhance both its workability and strength, thereby offering a viable solution^5^. Kuo’s experiments proposed that replacing a portion of river sand with shells in cement can lead to an increase in the proportion of shells. This substitution has had a notable impact on the overall compressive strength and shrinkage of the base material^6,7^. Eziefula has found that shells can be used as a partial or total substitute for fine and coarse aggregates in concrete^8^.

Some scholars in China used shells as aggregates to test concrete and found that the compressive strength of concrete increased with the rise in shell replacement rate after 28 days^9^. Sergey’s study showed that shells improved the strength, strain, modulus, and microstructure of concrete. The new concrete also reduced the cost and defects of construction. This research has practical implications^10^. Belarus researchers tested to use of agricultural waste (e.g., rice husk ash, peanut shells, oak wood chips, coconut shells, and corn cobs) as aggregate substitutes. This can lower waste pollution to the environment and construction costs^11,12^. Many studies have tested shells in concrete. From those tests, shells have more advantages over other agricultural wastes as concrete aggregates. Martínez found that shells should not exceed 25% for fine aggregates and 12.5% for coarse aggregates to improve concrete strength^13^. Malaysian researchers have suggested that Calcined shells improve concrete strength and density. Future research should test different calcination levels and shell concrete durability^14^. In addition, the research of Iraqi scholars reveals that crushed walnut shells(similar to shells) are an agricultural waste material that can be used to produce environmental concrete that reduces the demand for natural aggregates and the environmental impact of concrete production^15–17^.

After a thorough review of the above studies, it can be concluded that shells possess exceptional mechanical properties^18^, a significant specific surface area, and a lower density compared to stone. As a building material, shells offer advantages like workability, strength, and reduced weight in concrete^19^. To prepare the shells, they should be soaked in a concentrated solution of NaCl and a diluted solution of 10% H_2_O_2_^20^, after removing organisms and impurities, the material is crushed and screened to produce shells with a particle size of less than 0.5 mm. This not only helps save resources and energy but also reduces waste and decreases the consumption of natural aggregates^21,22^. As a result, it creates greater economic and social benefits^7,9,23^. Additionally, the study suggests that using the right amount of shell aggregate can enhance the strength of concrete, ensuring the safety and stability of structures while promoting sustainable development^24^.

Previous studies have shown that shells have adequate strength and some superior properties as aggregates^13^. However, there is no consensus on the best way to use shells in construction, and few practical examples exist. Herein, a research plan was proposed to test three levels of calcined shells (10%, 30%, and 50%) as aggregate replacements and compare their mechanical, economic, and environmental performance with conventional concrete. Our results confirmed that there’s an enhancement in maximum flexural strength and maximum compressive from these three-level shell aggregated concrete.

## Experimental program

This experiment uses different proportions of shells as concrete aggregate and prepares cement mortar according to a certain mix ratio and curing conditions. Different test equipment and methods are performed to measure the compressive strength, flexural strength, and water absorption rate with the purpose of studying the effect of shell replacement of crushed stone on the performance of cement mortar.

### Experimental materials and proportions

#### The performance of the shell

Shells are the hard protective coverings of various mollusks, such as oysters, clams, mussels, and scallops. They are composed mainly of calcium carbonate, also known as limestone, which is a common ingredient in cement^19^. Shells have a complex hierarchical structure, consisting of different layers of organic and inorganic materials, arranged in various patterns and orientations. This gives them high strength and toughness, as well as resistance to fracture and damage^25^.

The properties of seashell concrete depend on several factors, such as the type, size, shape, and proportion of seashells, as well as the curing conditions, admixtures, and chemical treatments. Some of the effects of seashells on concrete are:

Setting time: Seashells can increase the setting time of concrete, due to their alkaline nature and water absorption capacity^25,26^.

Workability: Seashells can decrease the workability of concrete, due to their irregular shape and rough surface^25^.

Density: Seashells can increase the density of concrete, due to their higher specific gravity than cement or sand^25,26^.

Compressive strength: Seashells can decrease the compressive strength of concrete, due to their lower bonding strength with cement paste and higher porosity 234. However, adding admixtures or applying chemical treatments can improve the compressive strength of seashell concrete^26,27^.

Tensile strength: Seashells can increase the tensile strength of concrete, due to their fibrous structure and crack-bridging effect^13,25^.

Flexural strength: Seashells can increase the flexural strength of concrete, due to their higher modulus of elasticity and toughness^26^.

Modulus of elasticity: Seashells can decrease the modulus of elasticity of concrete, due to their lower stiffness and higher deformation^27^.

#### Experimental materials

Cement: P·O R45 cement, the main chemical composition is presented in Table 1, while the physical and mechanical properties are displayed in Table 2, which complies with the requirements of EN 197-1 for chemical composition, strength, setting time, soundness, and fineness.

**Table 1:** The main component of cement %.

CaO	SiO2	Al2O3	Fe2O3	SO3	Na2O	K2O	MgO
64.12	22.31	6.42	4.37	1.1	0.75	0.56	0.37

**Table 2:** Physical and mechanical properties of cement.

Standard consistency water requirement/%	Specific surface area/(m^2^/kg)	Coagulation time/min		Compressive strength/MPa		Flexural strength/MPa	
		Initial setting	finalization	3 days	28 days	3 days	28 days
28	360	175	235	27.5	49.0	5.5	8.0

Water: ordinary tap water.

Sand: natural river sand, in line with the construction sand standard, the bulk density is less than 1.5 g/m^3^, the fineness modulus is 1.9, and the moisture content is less than 1%.

Stone: fine stone meets the requirements of EN 12620 for geometrical and physical properties, the bulk density is not less than 2.6 g/m^3^, and the particle size is 5–7 mm.

**Figure 3:**
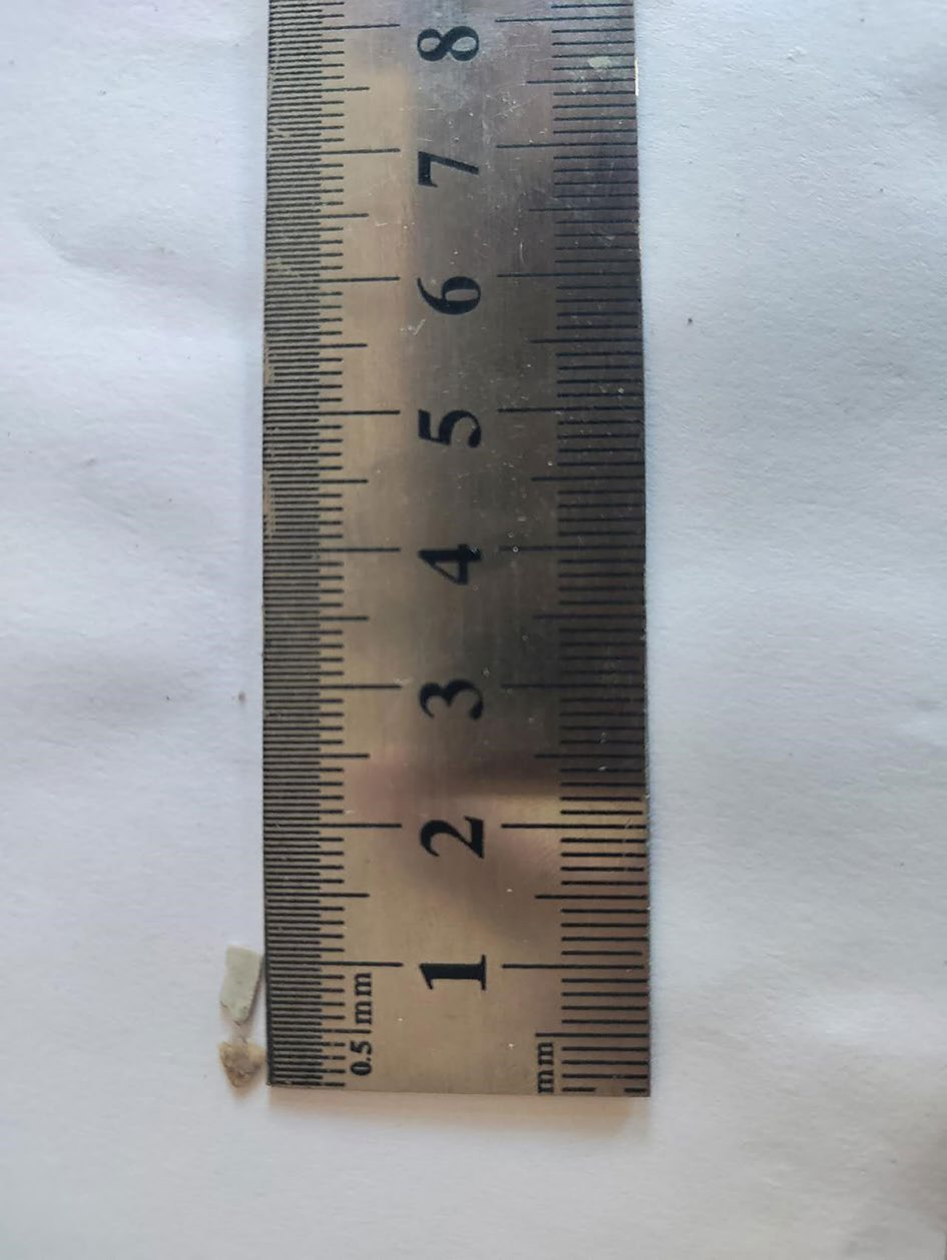
Shell particle size.

**Figure 4:**
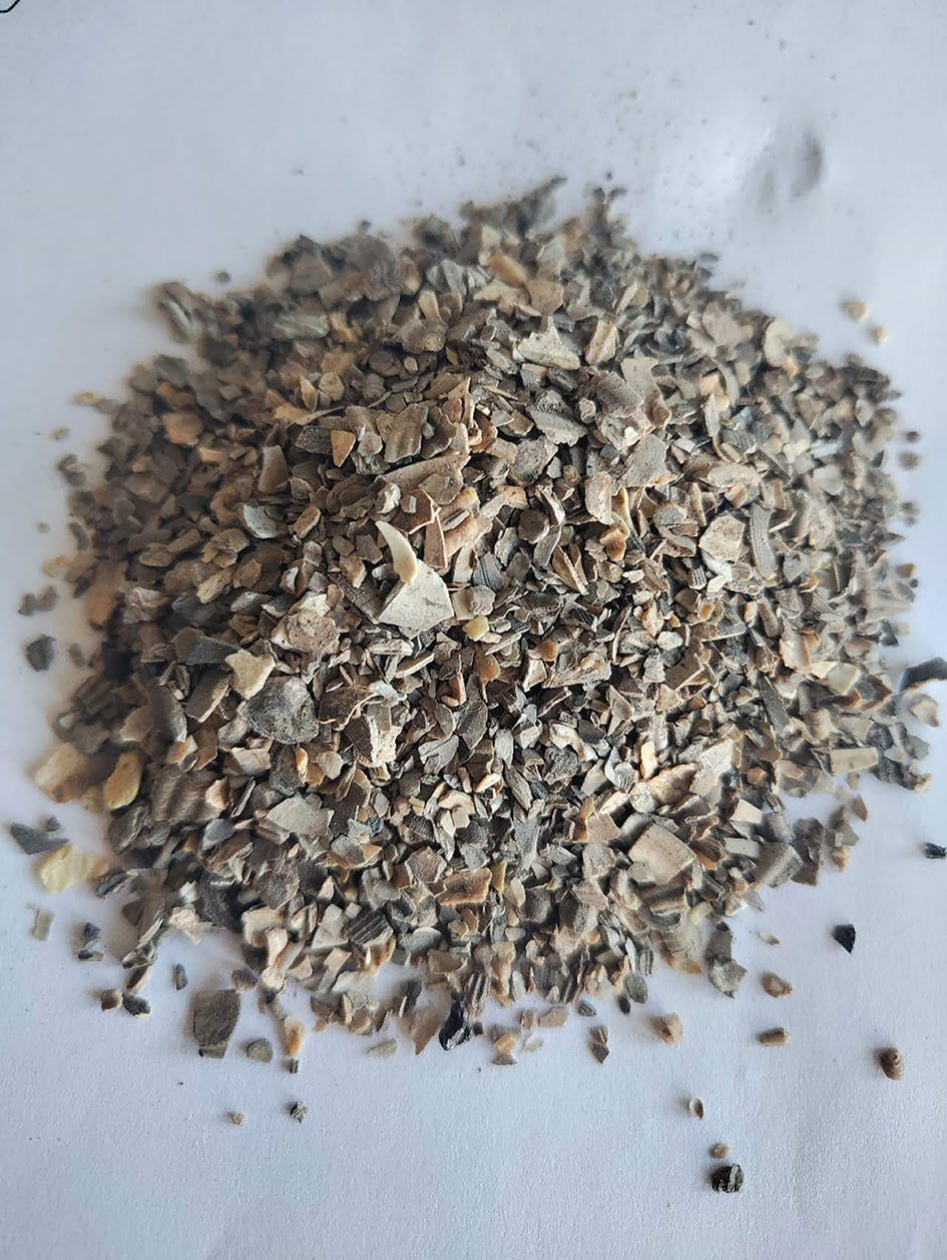
Shell accumulation.

Shell: According to the European Standards (EN-12620), the aggregates used in the production of concrete are inert granular materials such as gravel, crushed stone, sand, slag, recycled concrete, and geosynthetic aggregates. The aggregates may be natural, manufactured, or recycled, hence, the substitution of shells for aggregate meets the standards. After calcination and crushing, the particle size is less than 0.5 mm, the bulk density is less than 2.9 g/m^3^, the fineness modulus is 2.9, and the moisture content is less than 1%, refer to Figs. 3 and 4 for details.

The compressive strength of the concrete samples was tested at 7 days and 28 days after casting, according to EN 12390-3. The average compressive strength at 7 days was 25 MPa, 35 MPa on 28 days, which met the design requirement of 30 MPa for the structural elements.

The slump test was performed on the fresh concrete mix, according to EN 12350-2. The slump value was 75 mm, which indicated a medium workability of the concrete, suitable for the casting and compaction methods used in this project.

#### Material ratio and specimen design

**Figure 5:**
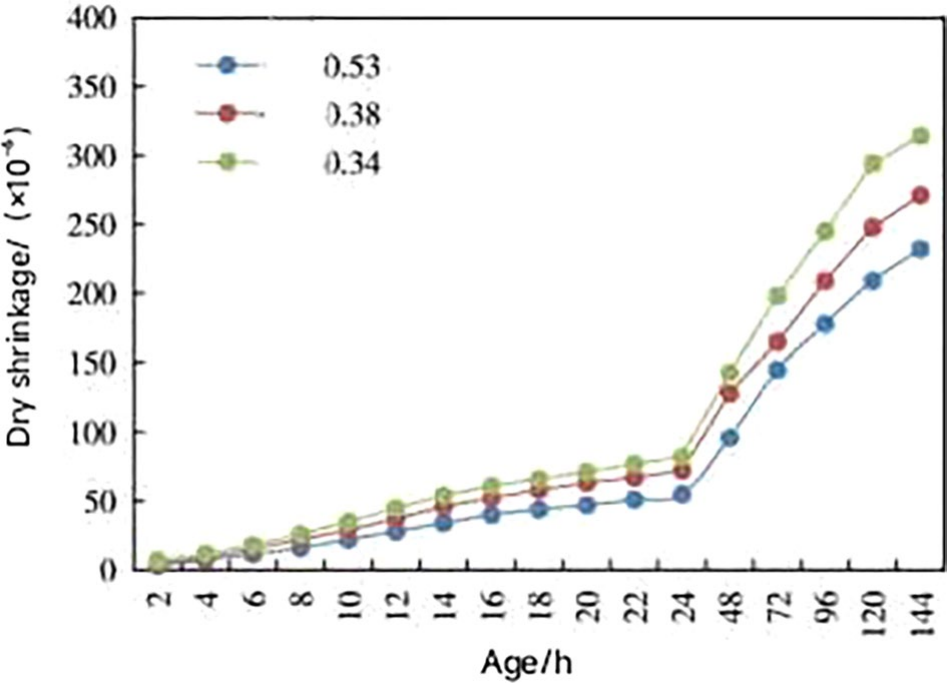
Dry shrinkage curves of concrete under different water cement ratios^9^.

**Figure 6:**
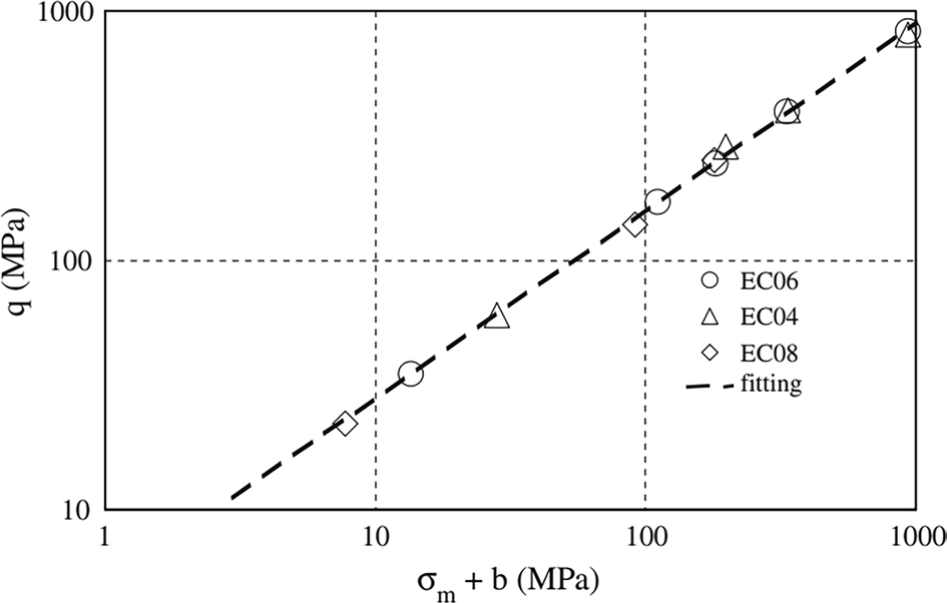
Water cement ratio and strength relationship curve^29^.

According to the study’s conclusions and curve analysis, it is believed that, as the water-binder ratio increases, the impact of coarse aggregate becomes more pronounced, resulting in lower dry shrinkage^28^, referring to Fig. 5. Previous studies also show an increasing the water-cement ratio results in higher porosity and lower strength of the concrete^29^. After analyzing Figs. 5 and 6, and considering EU standard EN 206+A2 and Chinese standard GB 50010-2010, the water-cement ratio in the range of 0.45–0.6 is relatively appropriate^30^, to ensure sufficient strength and reduce porosity, it is necessary to choose a smaller water-cement ratio^31^. So our team take a water-cement ratio of 0.5 as optimal for ensuring the strength and preventing cracking of the concrete in the experiment^31,32^.

**Figure 7:**
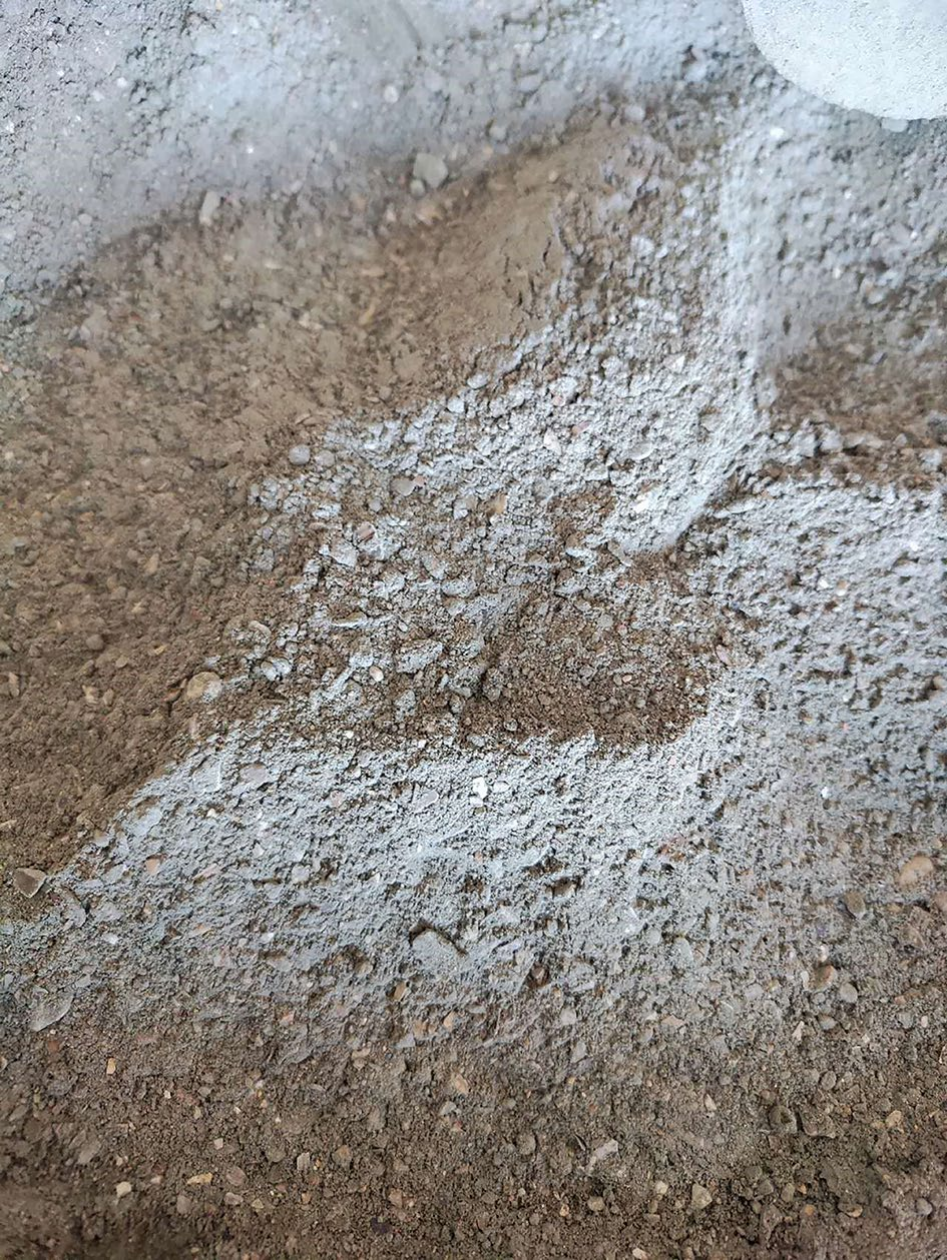
Mixture.

**Figure 8:**
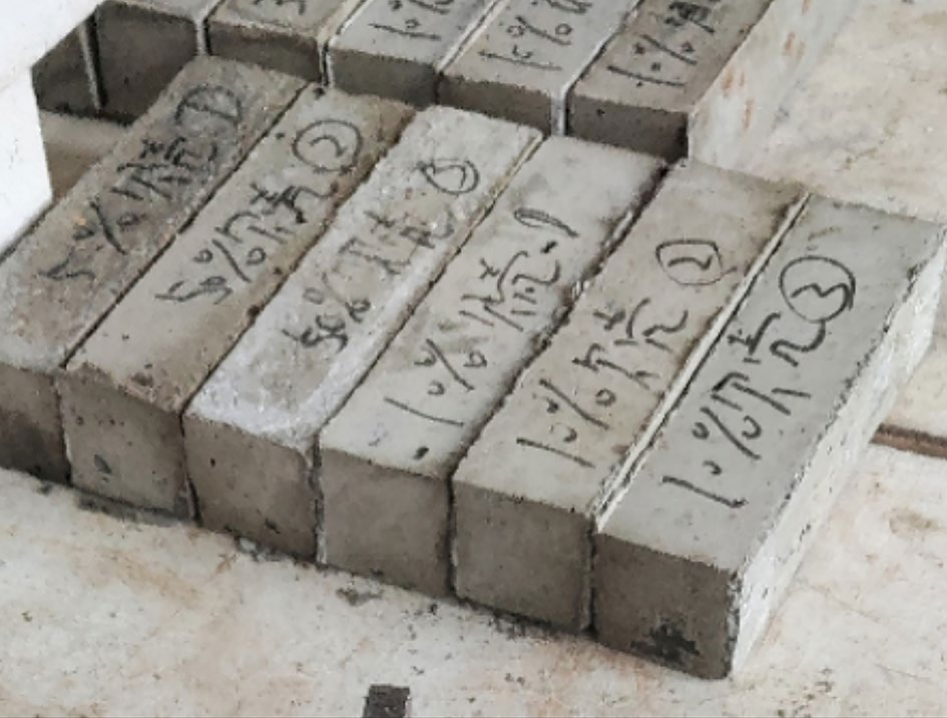
Test block sample.

In this test, shells and other materials were used as a substitute for natural stone. The mix ratio (kg/m^3^) of the cement mortar in the benchmark group was as follows: m (cement): m (sand): m (stone): m (water) = 500: 600: 900: 250, the mix ratio (kg/m^3^) of the cement mortar in the 10% shell replacement rate was as follows: m (cement): m (sand): m (stone) : m(shell): m (water) = 500: 600: 810:90: 250, the mix ratio (kg/m^3^) of the cement mortar in the 30% shell replacement rate was as follows: m (cement): m (sand): m (stone) : m(shell): m (water) = 500: 600: 530: 270: 250, the mix ratio (kg/m^3^) of the cement mortar in the 50% shell replacement rate was as follows: m (cement): m (sand): m (stone) : m(shell): m (water) = 500: 600: 450: 450: 250. All the mixes conform to standard EN 206-1, the mixture is shown in Fig. 7. Additionally, the superplasticizer content was 0.2% of the cement mass. The gravel is replaced based on shell gradients of 10%, 30%, and 50%. The mixture was evenly stirred and poured into a mold with dimensions of 400 mm × 400 mm × 1600 mm, the test prisms is shown in Fig. 8, following standard EN 12,390–5:2009. Thirteen specimens are cast for each different gradient, 39 specimens in total for bending experiments. The specimens for compressive experiments are 100 mm × 100 mm × 100 mm, following the standard EN 12390-3:2019, with 13 specimens cast at each gradient, 39 specimens in all. After segmental vibration compaction, the specimens were hardened at 23 °C, molded after 7 days, and cured at 20 °C with a relative humidity of 95% until the specified age^33^.

#### Experimental procedure

After curing for 28 days, the specimen underwent a test using the DS2-1000N compressive strength tester for axial compressive resistance and three-point bending. The “Hydraulic Pressure Testing Machine-GB/T3722” was used to conduct a uniaxial compressive test on the specimen, aligning the axis with the pressure center of the testing machine's pressure plate. The load was applied at a speed of 10–30 kN/s until the specimen failed, and the failure load was recorded to determine the compressive and flexural strengths^33^. The test block was soaked in water for 2 days, taken out to dry completely, recorded the weight before and after. The water absorption rate was calculated to evaluate the frost resistance strength.

## Experimental results and analysis

### Flexural strength

**Figure 9:**
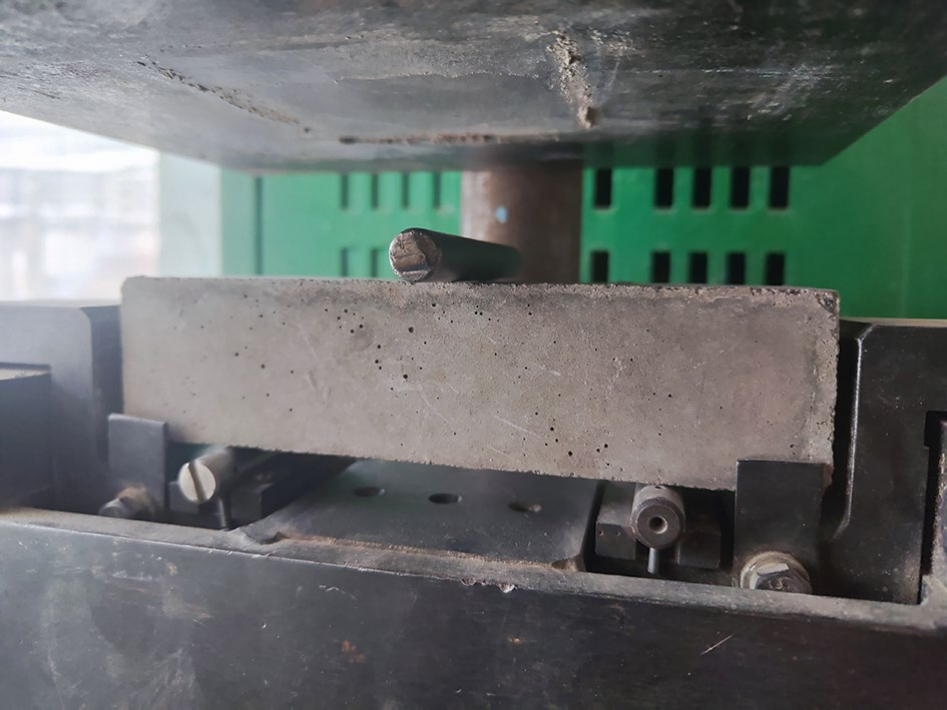
Flexural test.

**Figure 10:**
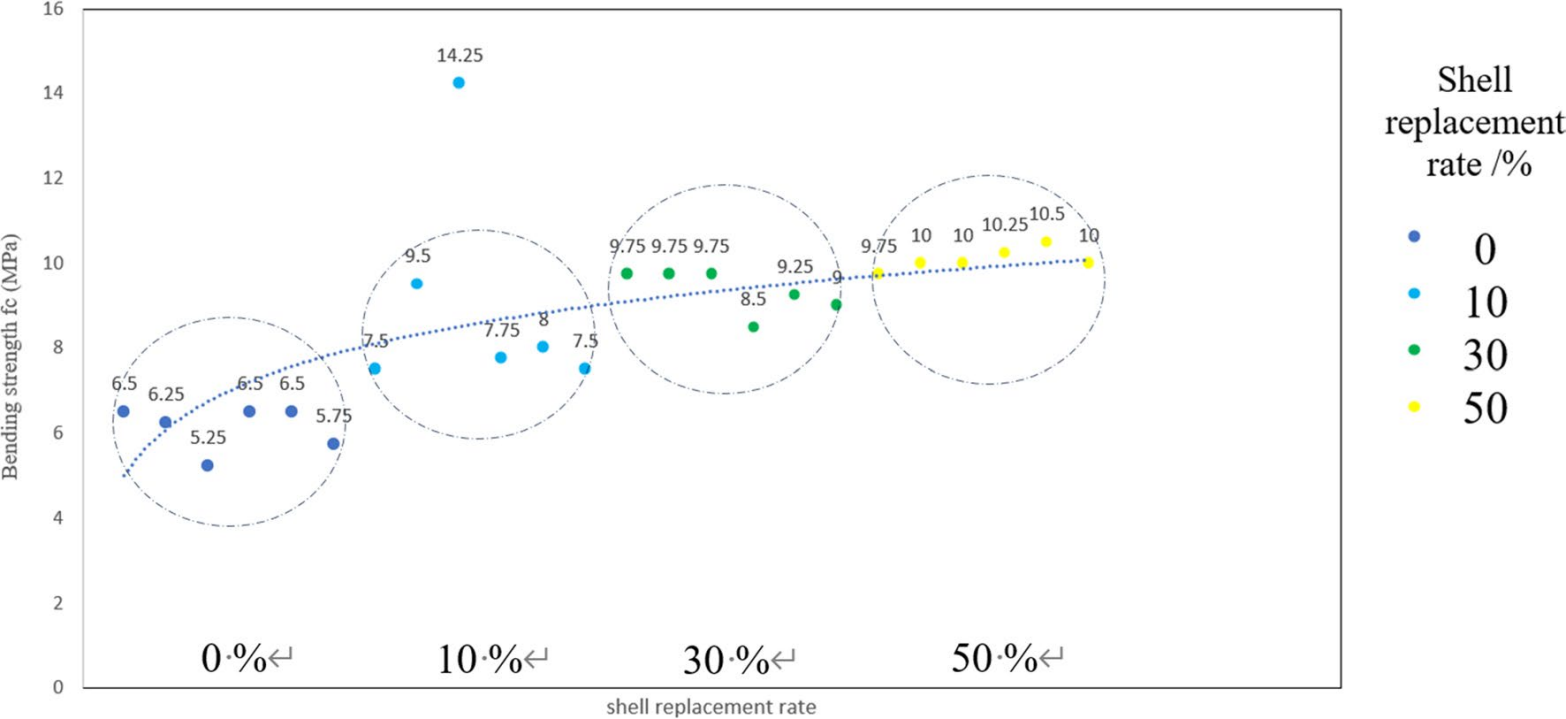
Scatter plot of flexural strength.

The failure load of the specimen was obtained through a three-point bending test, testing followed the third-point loading because it is simpler to perform and analyze than the fourth-point loading^34^. This testing was following the standard ASTM C78/C78M and the flexural strength is presented in Table 3, and the flexural strength ff (MPa) of the specimen is calculated according to the formula ${\varvec{f}}_{{\varvec{f}}} = \frac{{{\varvec{Fl}}}}{{{\varvec{bh}}^{2} }}$, the testing is shown in Fig. 9, where ff is the concrete flexural strength (MPa), F is the failure load of the specimen (N), L is the span between the supports (mm), and b is the cross-sectional width of the specimen (mm), h is the cross-sectional height of the specimen (mm); the scatter plot distribution of flexural strength is shown in Fig. 10.

**Table 3:** Concrete flexural strength under different substitution rates.

Shell replacement rate/%	Flexural strength f_f_ (MPa)					
0 (Standard Group)	6.5	6.25	5.25	6.5	6.5	5.75
10	7.5	9.5	14.25	7.75	8	7.5
30	9.75	9.75	9.75	8.5	9.25	9
50	9.75	10	10	10.25	10.5	10

### Compressive strength

**Figure 11:**
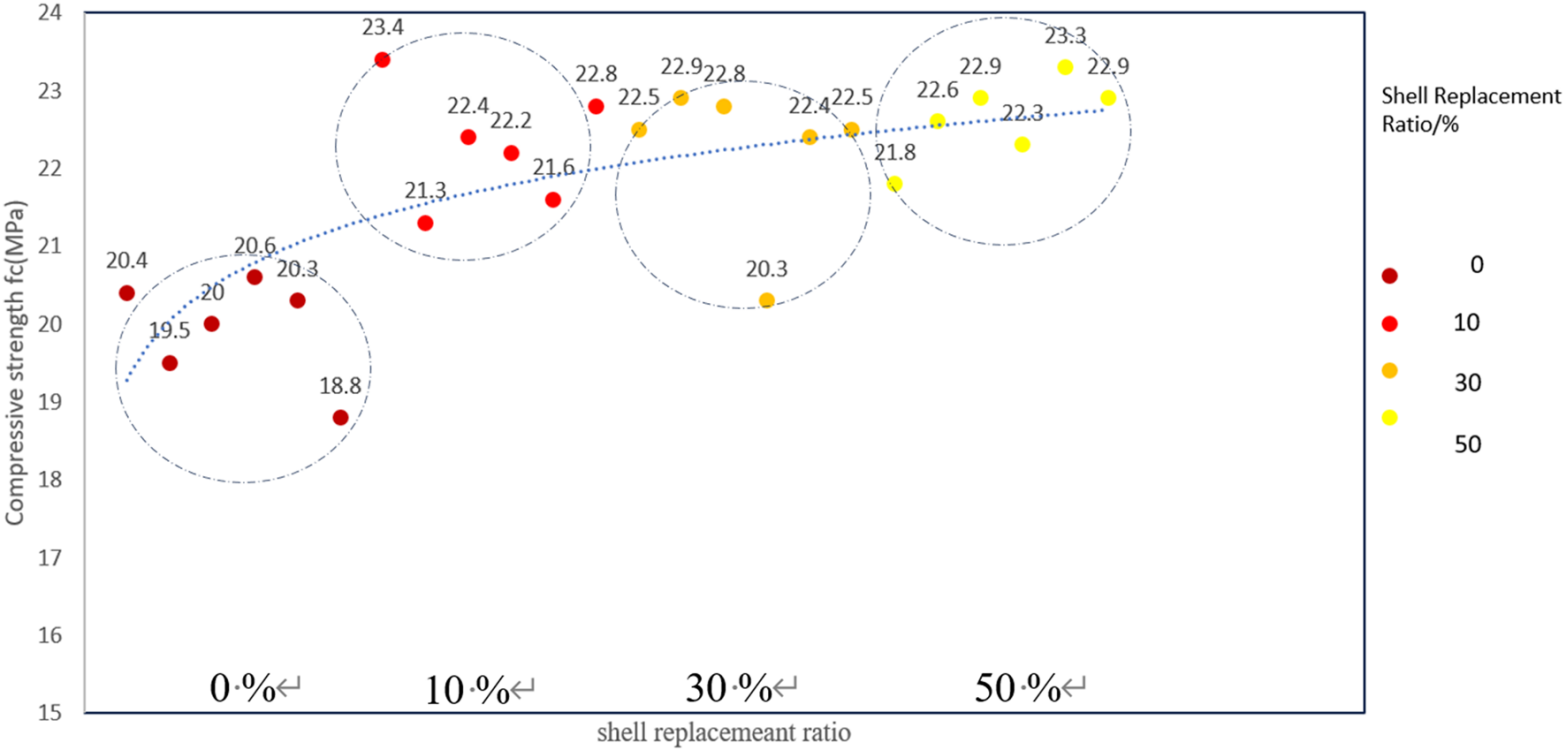
Scatter plot of compressive strength.

**Figure 12:**
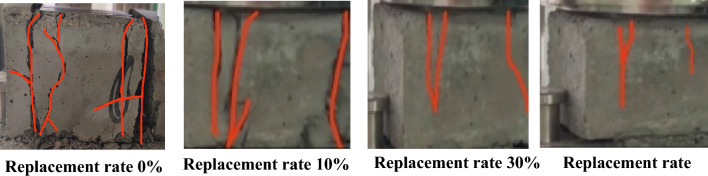
Destruction tracking.

Throughout the uniaxial compressive test, the uniaxial compressive failure load of the specimen is determined, and the compressive strength ${\varvec{f}}_{{\varvec{c}}}$ (MPa) of the test cube can be calculated according to the formula ${\varvec{f}}_{{\varvec{c}}} = {\varvec{F}}/{\varvec{A}}$, where ${\varvec{f}}_{{\varvec{c}}}$ is the compressive strength, F is the failure load, and A is the bearing area. Intensity data is presented in Table 4. The scatter plot distribution of compressive strength is shown in Figs. 11. The damage situation is depicted in Fig. 12.

**Table 4:** Compressive strength of concrete under different replacement rates.

Shell replacement ratio/%	Compressive strength fc (MPa)					
0 (Standard Group)	20.4	19.5	20	20.6	20.3	18.8
10	23.4	21.3	22.4	22.2	21.6	22.8
30	22.5	22.9	22.8	20.3	22.4	22.5
50	21.8	22.6	21.9	22.3	23.3	22.9

### Microstructure analysis

**Figure 13:**
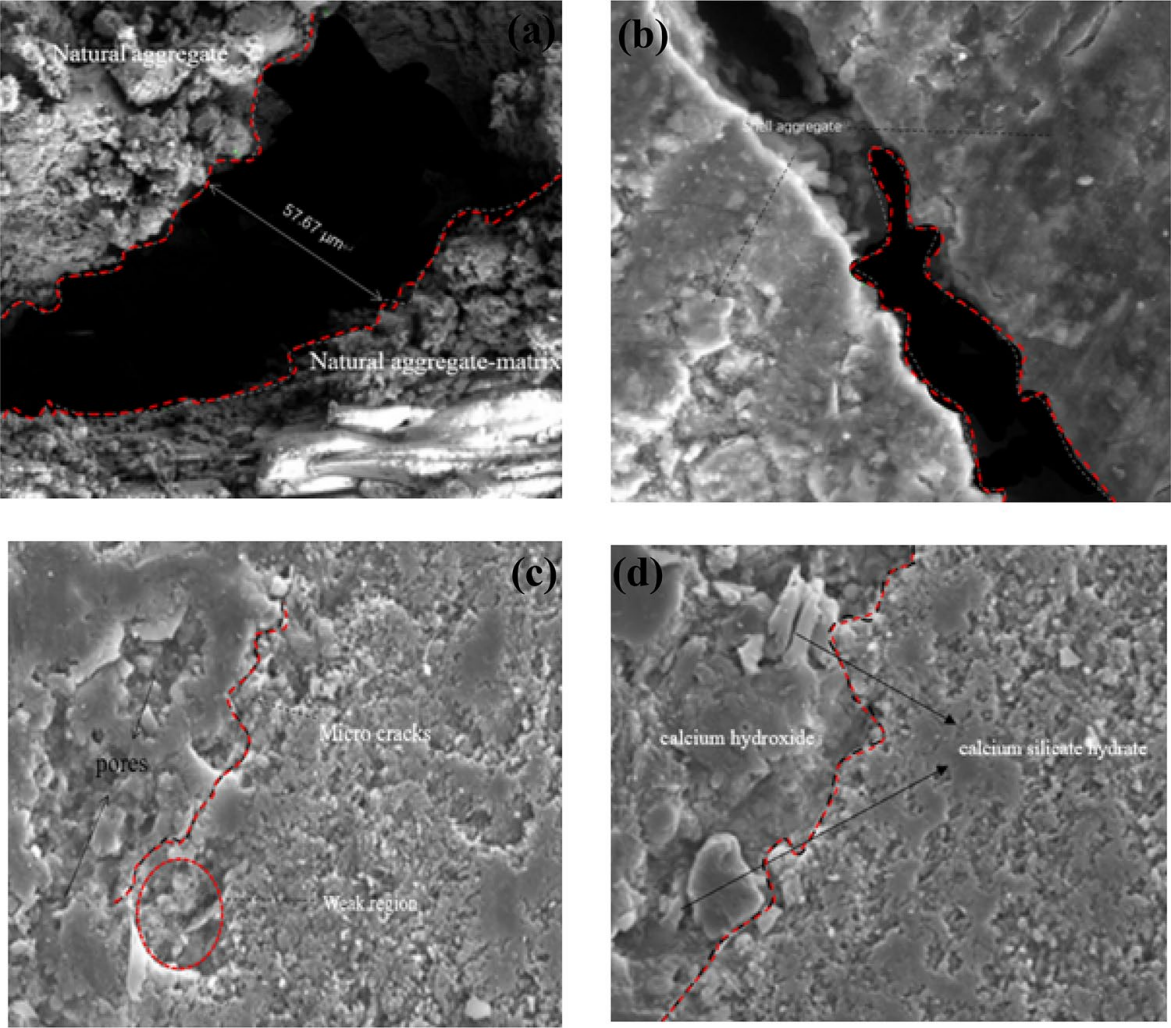
Results of SEM tests.

To carry out a more comprehensive analysis of the impact of shell aggregates on the mechanical properties of concrete at a microscopic level, scanning electron microscopy (SEM) was performed on shell aggregates with varying replacement rates (0%, 10%, 30%, and 50%). This allowed us to observe the surface structure of the concrete under different shell replacement conditions. Figure 13a depicts concrete without shell aggregate, and Fig. 13b illustrates concrete with a 10% shell aggregate. In Fig. 13c, concrete with a 30% shell aggregate is shown, and Fig. 13d displays concrete with a 50% shell replacement rate. Comparing these images, it is evident that the inclusion of shell aggregate in concrete enhances the compactness of the joints between concrete structures, resulting in improved stress performance and a reduced risk of compressive cracking. The overall working performance of the concrete is enhanced.

### Waters absorption ratio

**Figure 14:**
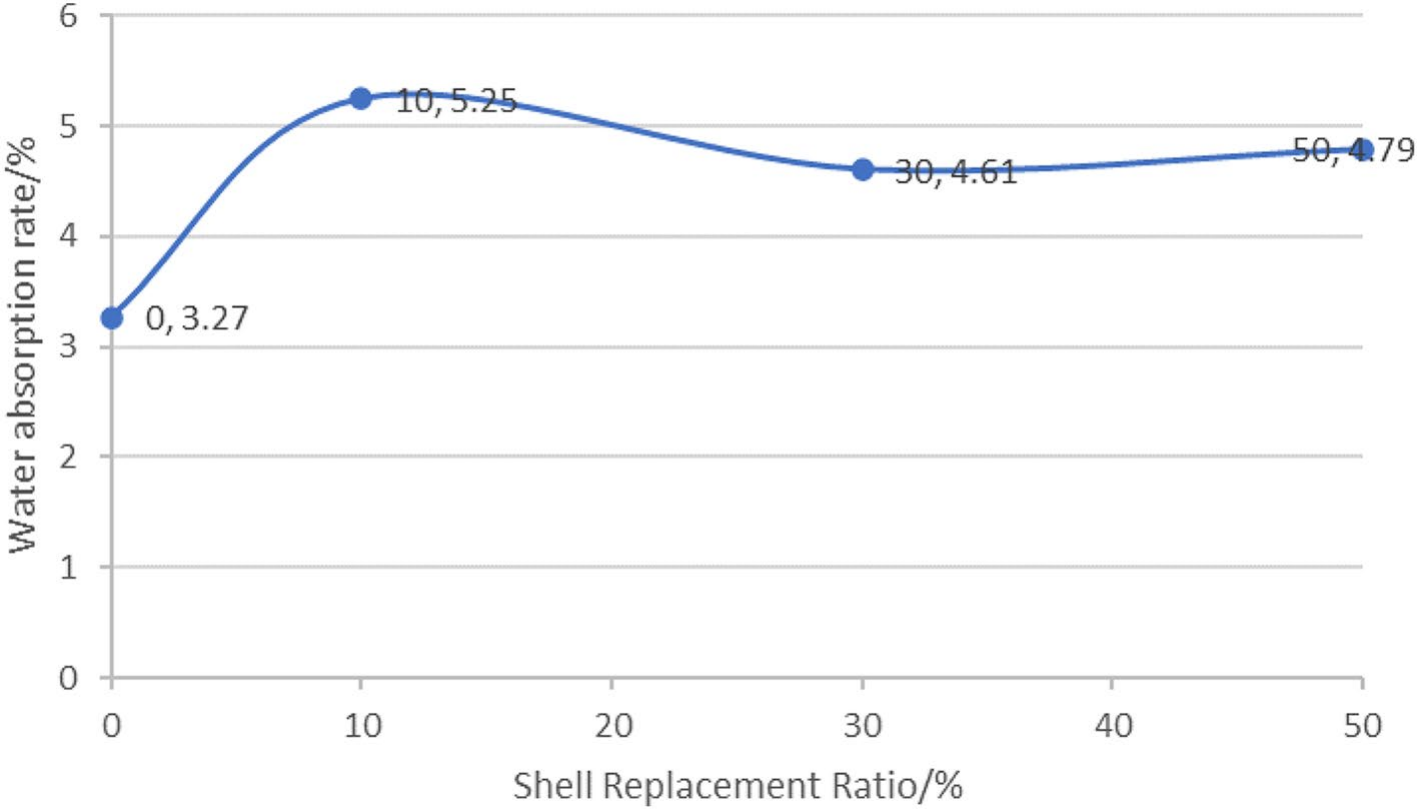
Water absorption rate.

After measuring the mass of the samples before and after, the water absorption rate can be calculated by the formula ${\varvec{W}} = \left( {{\varvec{B}} - {\varvec{G}}} \right)/{\varvec{G}} \times 100\user2{\% }$, G is the weight of the sample after drying, B is the weight of the sample saturated with water, the testing method was according to EN 12390-8:2009, and the data is presented in Table 5. The curve depicting the change in water absorption is shown in Fig. 14.

**Table 5:** Concrete moisture content under different substitution rates.

Shell replacement ratio/%	Weight before tumbling/g	Weight after drying/g	Water absorption/%
0 (Standard Group)	634.5	614.4	3.27
10	607.1	576.8	5.25
30	609.8	582.9	4.61
50	601.2	573.7	4.79

## Prediction of the mechanical strengths

Based on the analysis of the data obtained from the three-point flexural test (Table 3), the following conclusions can be drawn:

- When the shell replacement rate is 10%, the flexural strength of the specimen increases by 31.4% and 1.925 MPa compared to the benchmark group.
- When the shell replacement rate is 30%, the flexural strength of the specimen increases by 52.4% and 3.208 MPa compared to the benchmark group.
- The flexural strength of the specimen increases by 64.6% and 3.958 MPa, respectively, when the shell replacement rate is 50%.

According to the mechanical curve shown in Fig. 10, it is evident that the addition of the shell significantly enhances the flexural strength of the prism when compared to traditional aggregate. This indicates that the inclusion of shell aggregate reduces the concrete's susceptibility to bending and fracturing. Consistent with the experimental phenomenon, previous research has shown that the addition of shells improves post-cracking behavior, reduces the opening of cracks, and counteracts their expansion, as well as increases the toughness of concrete owing to their deboning and internal stress mechanisms^35,36^. Furthermore, due to the adsorption capacity of shells^37^, if the shell is aligned with the tensile direction, it is capable of bearing the tensile force and impeding crack propagation^38^. Therefore, the shells with higher content have a wider and more uniform distribution in the concrete, as the cement does not have a significant effect on the shells during the hydration process, and no settlement or floating occurs, which also improves the tensile strength of the concrete. These findings are supported by the available data, when the shell replacement rates were 10%, 20%, and 30% respectively, the mixing ratio increased by 10% each time, and the 90 d tensile strength of the concrete with traditional aggregate increased by 11.8%, 15.1%, and 17.6% respectively^6,8,25,35^.

In the uniaxial compressive test (Table 4), it was observed that the compressive strength of the specimen increased by 10.7% and 2.727 MPa when the shell replacement rate was 10% compared to the benchmark group. Similarly, when the shell replacement rate was 30%, the compressive strength of the specimen increased by 16.6% and 3.207 MPa compared to the benchmark group. The compressive strength of the specimen increased by 17.8% and 3.442 MPa compared to the benchmark group when the shell replacement rate was 50%. The curve depicted in Fig. 11 shows that the inclusion of shell aggregates leads to a significant enhancement in compressive strength. This observation suggests that the addition of shell aggregates improves the concrete's resistance to compression-induced damage and enhances its load-bearing capacity. Upon comparing the curves depicted in the Figs. 10 and 11, it is evident that the curve exhibits a rapid rise in the range of 0–30% replacement rate. However, the rate of increase slows down between 30 and 50% and eventually reaches a plateau when it approaches 50%. This observation suggests that shells can be substituted at a rate as high as 50% while still providing satisfactory strength as aggregates.

Figure 13 presents scanning electron microscope (SEM) images of shell concrete at a 28-day scale, depicting various shell aggregates with distinct gradients in intensity. Based on the captured images of moral onlookers, a further analysis was conducted on the impact of shell aggregates on the mechanical properties of natural aggregates. It can be observed from the SEM diagram of the failed samples that the cracking in the shell aggregate concrete with 0% content is more pronounced and the spacing between cracks is larger. In contrast, the shell aggregate concrete with 10% content exhibits significantly smaller fracture spacing compared to the 0% content. Moreover, the shell aggregate concrete with 30% and 50% content only shows a limited number of microcracks and pores, which can be considered mechanically weak areas. Hence, it has been demonstrated that incorporating crushed shells into concrete under identical pressure conditions can effectively enhance the mechanical properties of the concrete. Further examination of Fig. 13b–d reveals that the concrete structure exhibits a high level of compactness, with no presence of a cementitious porous structure. This observation effectively demonstrates the favorable workability of the concrete material when shell aggregate is added as a composite material. Additionally, the shell aggregate is capable of forming a dense matrix structure with the natural aggregate, resulting in strong compactness and low porosity. This further enhances the bonding between the cement slurry and the aggregate matrix. Therefore, the incorporation of shell aggregate as a partial replacement for natural aggregate has been found to enhance the mechanical properties of concrete significantly.

Cong proposed that there was a positive correlation between the moisture content of concrete and its higher frost resistance^39^. Therefore, it is hypothesized that the frost resistance of the test block may be influenced by its water absorption capacity, it can be considered that the lower the water absorption rate, the more favorable the frost resistance of the material^40^. It is expected that a lower water absorption rate would result in lower moisture content within the test block, thereby enhancing its frost resistance^41^. Based on the analysis of the curves presented in Table 5 and Fig. 8, it can be inferred that the replacement of traditional aggregate with shell leads to a slight increase in the moisture content of the test block. It can be concluded that the frost resistance of the shell aggregate is poor. With the gradual increase in the replacement rate of aggregate, there is minimal fluctuation in the moisture content. It can be inferred that while the replacement rate of shell aggregate may decrease the frost resistance, varying replacement rates have a negligible impact on the frost resistance of concrete.

Furthermore, in the process of the experiment, it was observed that the application of mechanical vibration in the concrete exhibited a flow-like behavior, allowing it to fill the formwork evenly and densely. The destruction section displayed a uniform distribution of shells, with no instances of delamination or segregation. Additionally, it was noted that there were no significant issues with water leakage during the curing process. It can be inferred that the use of shells as aggregates exhibits favorable workability and integrity.

## Life cycle assessment

### Economical assessment

The researchers believe that in the process of studying the replacement of traditional aggregates by shells, to explore the application of aggregates in actual production, it is very important to analyze its economic performance. Take the China Construction Project Budget Quota to calculate the cost of concrete: 180 yuan/m^3^ for sand, 4.1 yuan/m^3^ for commercial water, 635 yuan/ton for cement, 220 yuan/m^3^ for stone, one cubic meter of concrete weighs 2400 kg. The cost of commercial concrete is 635 yuan/m^3^^42^. In addition, the researchers investigated the garbage recycling plant and recycled the waste shells, found that the waste shells were biological waste, and the recycling plant believes that they do not have recycling value, so they can be obtained free of charge, and only need to issue relevant labor costs, and the cost of shells can be calculated according to the labor cost of 10 yuan per m^3^^43^.

#### Economic performance analysis of buildings using frame shear structure

**Figure 15:**
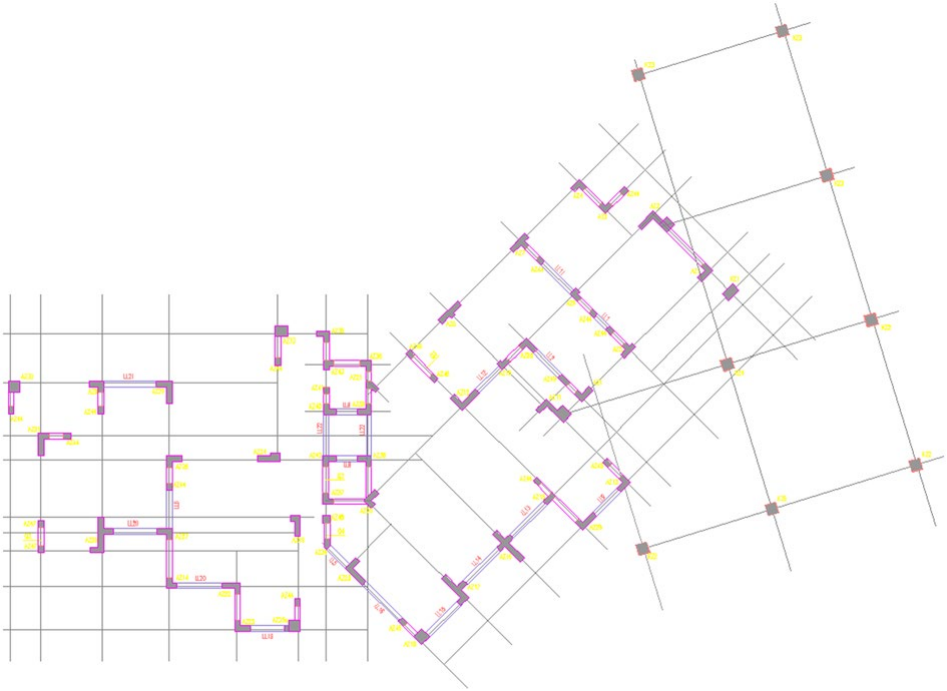
Frame-shear wall structure.

Here, a significant high-rise building project covering a total area of 173,256.37 m^2^ in the East of China is taken as a sample. The structure of the building is depicted in Fig. 15. To assess the economic viability of shell aggregates, all concrete materials were substituted with 50% shell aggregate concrete. Upon comparison with traditional aggregates, it was found that the total concrete consumption amounted to 82,226.37 m^3^. Full analysis details are given in Appendix 1.

#### Analysis of the economic performance of buildings using frame Structures

**Figure 16:**
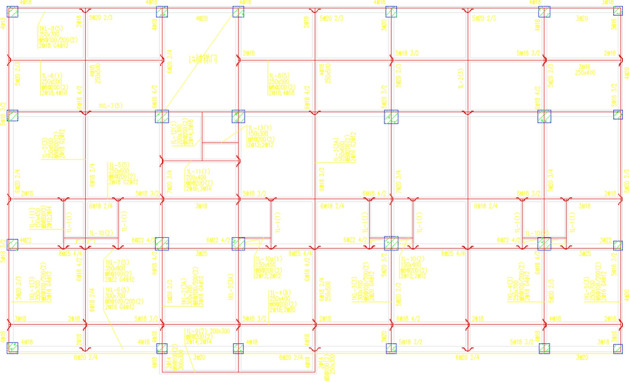
Frame structure.

A nine-story frame structure in southern China serves as a sample with a total area of 12,055.52 m^2^ (Fig. 16) to assess the economic performance of shell aggregates. An analysis was carried out by replacing all concrete with 50% shell aggregate concrete compared to traditional aggregates. The total concrete consumption for this structure amounted to 3389.92 m^3^. Full analysis details are given in Appendix 2.

#### Analysis of the economic performance of the building using the tube in the tube

**Figure 17:**
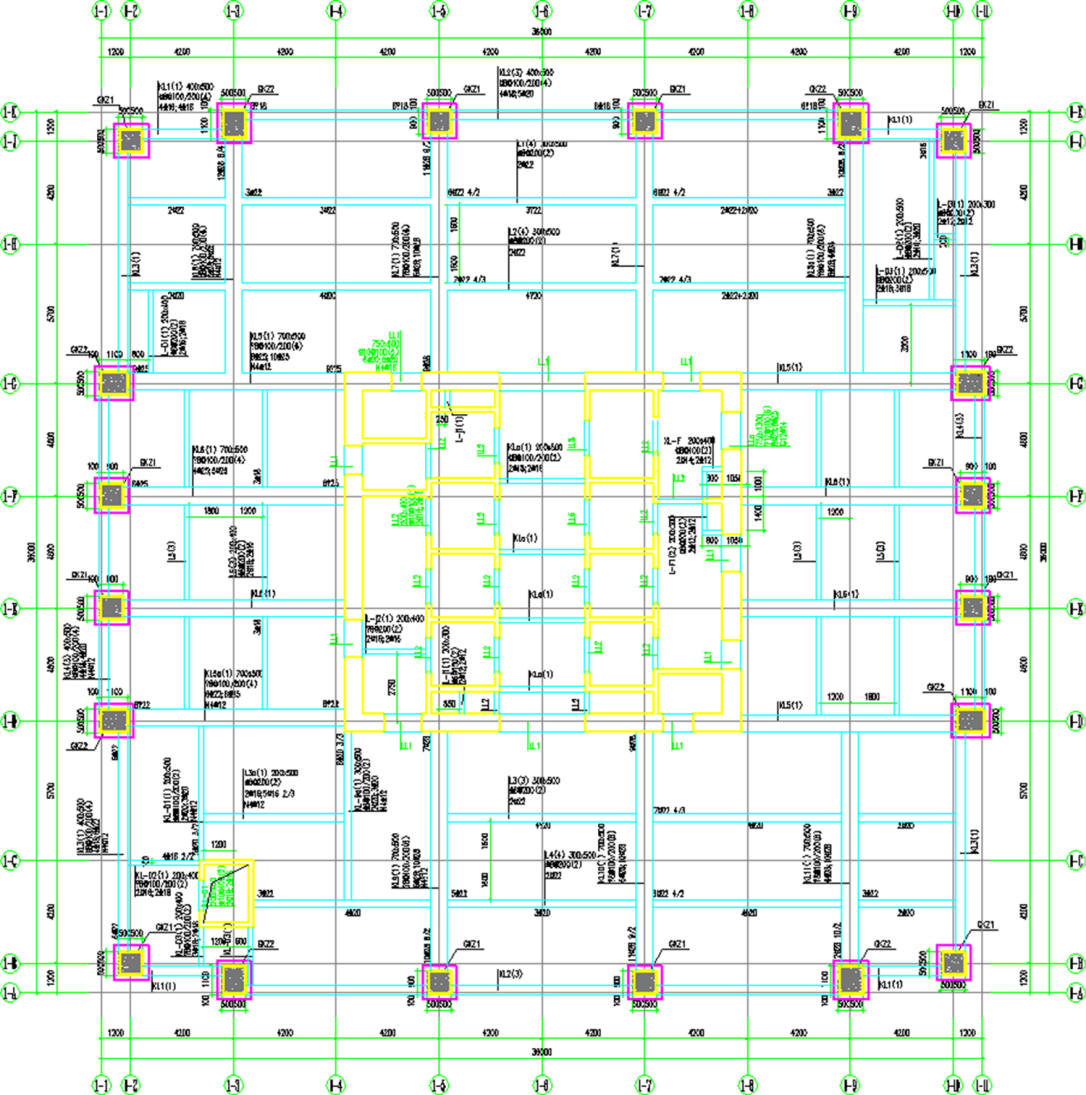
Tube in tube structure.

A building with a tube structure in southern China was put as a sample, as depicted in Fig. 17, which has a total area of 341,376.54 m^2^. To evaluate the economic performance of shell aggregate, an analysis is based on replacing all concrete with 50% shell aggregate concrete. The total volume of concrete used in this structure was 263,124.38 m^3^. Full analysis details are given in Appendix 3.

**Figure 18:**
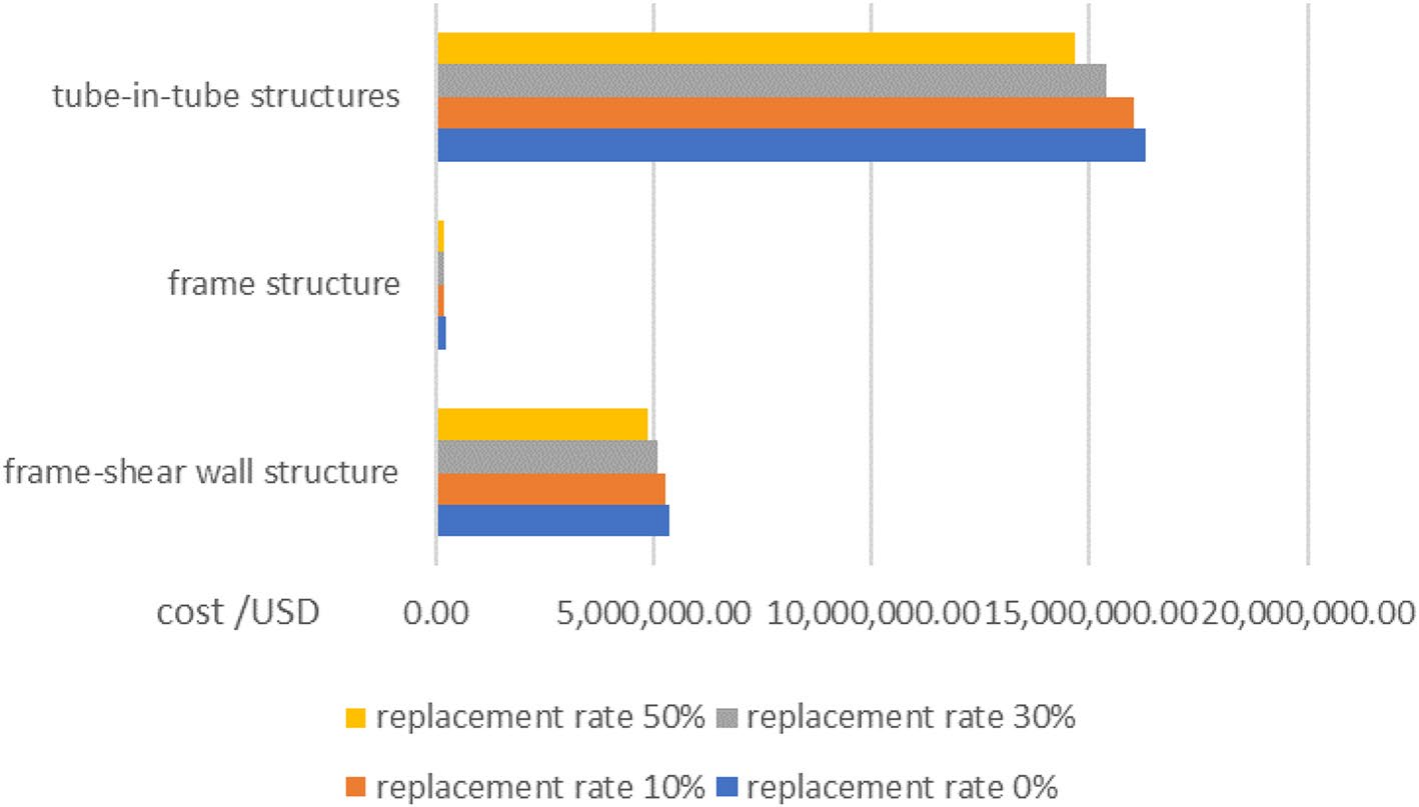
Cost comparison.

Compare the cost of replacing traditional aggregates with shells in three building structures (Fig. 18), the researchers calculated that the concrete costs of the frame-shear structure, frame structure, and tube-in-tube structure are reduced by 10.2%, 10%, and 10.3% respectively. This suggests that shell aggregate can effectively reduce building costs.

### Environmental assessment

To assess the environmental impact of shell aggregates, it is important to consider carbon emissions. This includes the carbon emissions from the production of raw materials (C_1a_), which refers to the CO_2_ generated during the production and processing of raw materials. This includes energy consumption and carbon emissions from the material processing itself. The calculation formula for C_1a_ is as follows:${\varvec{C}}_{{1{\varvec{a}}}} = \mathop \sum \limits_{{\varvec{i}}} \left( {\mathop \sum \limits_{{\varvec{j}}} {\varvec{a}}_{{{\varvec{ij}}}} {\varvec{K}}_{{\varvec{j}}} } \right){\varvec{m}}_{{\varvec{j}}} + {\varvec{g}}_{1} {\varvec{m}}_{1}$. In the formula, ${\varvec{a}}_{{{\varvec{ij}}}}$ represents the energy consumption of j in the production process of i raw materials. ${\varvec{m}}_{{\varvec{j}}}$ represents the amount of class I raw materials used in 1 m^3^ of recycled concrete. ${\varvec{K}}_{{\varvec{j}}}$. is the carbon emission coefficient of class J energy, which is the sum of the direct carbon emission coefficient ${\varvec{K}}_{{\varvec{j}}}$. and the indirect carbon emission coefficient ${\varvec{K}}_{{\varvec{j}}}$. ${\varvec{g}}_{1}$ represents the carbon emission generated by the material itself in the process of cement production, m_1_ represents the ount of cement in 1 m^3^ of recycled concrete^44^. The study will utilize the mixture ratio of experimental test blocks to calculate parameters for carbon emissions. The calculation parameters for carbon emissions are presented in Table 6, while Table 7 displays the carbon emissions resulting from energy consumption. Full analysis details are given in Appendix 4.

**Table 6:** Carbon emission calculation parameters^44,45^.

Unit material	Power consumption/(kW h)	Coal consumption/Kg	Diesel consumption/L
1t cement	40	96	
1t natural coarse aggregate	1.17		0.723
1t sand	1.5		0.8
1t water	0.29

**Table 7:** Carbon emissions generated by energy /kg.

Unit energy	Direct carbon emissions	Indirect carbon emissions	Total carbon emissions
1 kW h of electrical energy	0	1.195	1.195
1 kg of coal	2.53	0.088	2.618
1L diesel	2.73	0.448	3.178

**Figure 19:**
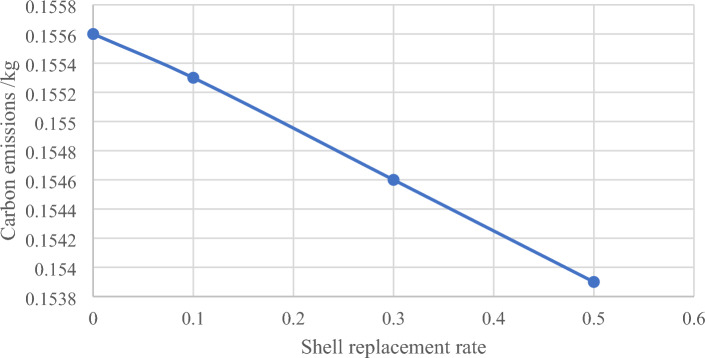
Carbon emission curves of concrete under different replacement rates.

The carbon emission curve of concrete at different shell replacement rates suggests that incorporating shells, as shown in Fig. 19, can effectively reduce carbon emissions. This reduces energy consumption and minimizes the environmental impact of buildings, protecting the ecological environment^46^.

## Conclusion

To solve the bio-waste recycling problem, the aggregate approach method is proposed and tested on the mechanical properties of the concrete specimens with 10%, 30%, and 50% crushed bio-shell as a replacement aggregate. Three different types of buildings are calculated the concrete cost as an example and the carbon environmental impact of the specimens is evaluated using a carbon environmental protection formula.

Our result has shown that the substitution rate of shell can reach up to 50%, and the higher the substitution rate within this range, the greater the enhancement in concrete strength. The maximum increase in flexural strength can reach 3.96 MPa, and the highest increase in compressive strength can reach 2.5 MPa. Calculation of the project’s actual content found that using shell aggregate concrete can reduce material costs by approximately 10% for various structural projects. This significantly addresses the issue of excessive project costs. Utilizing a formula focused on carbon environmental protection, it has been determined that shell aggregate demonstrates notable environmental benefits. Moreover, this method significantly reduces kitchen waste, lowers energy usage, curtails the ecological footprint of carbon dioxide emissions, improves the overall ecosystem, and consequently delivers substantial societal advantages.

Overall, this work proves that aggregate concrete matrix can be a good method to reduce the waste of biohazards. A few questions can be raised from data analysis like if we can optimize the structure of the concrete or if we can find a better mix ratio when adding more types of bio-waste. Future research ideas will based on these unanswered questions.

### Supplementary Information

Supplementary Information 1.

Supplementary Information 2.

Supplementary Information 3.

Supplementary Information 4.

## Data Availability

Data is provided within the manuscript or supplementary information files.
