# Supplementary material for: Effect of biological shells aggregate on the mechanical properties and sustainability of concrete
Source: Sci Rep. 2024 May 9;14:10615. doi: 10.1038/s41598-024-61301-1 (PMC11078922; doi:10.1038/s41598-024-61301-1)
Supplement: Supplementary file 1 — Supplementary Information 1. [file 41598_2024_61301_MOESM1_ESM.docx]

# Appendix 1

The ratio of raw concrete, as calculated based on the quota, is m (cement): m (sand): m (stone): m (water) = 5: 6: 9: 2.5.

cement 0635×5/(5+6+9+2.5)×82226.37×2400=27847330 yuan,

sand 180×6/(5+6+9+2.5)×82226.37=3946865 yuan,

stone 220×9/(5+6+9+2.5)×82226.37=7235920 yuan,

water 4.1×2.5/(5+6+9+2.5)×82226.37=37458 yuan,

so the total cost of concrete is 39,067,573 yuan, or 5,351,355.80 in US dollars.

The ratio of concrete aggregate, consisting of cement, sand, shell, stone, and water, is calculated as follows: m (cement) : m (sand) : m (shell) : m (stone) : m (water) = 5 : 6 : 4.5 : 4.5 : 2.5, based on the prescribed proportions:

cement 0635×5/(5+6+9+2.5)×82226.37×2400=27847330 yuan,

Sand 180×6/(5+6+9+2.5)×82226.37=3946865 yuan,

stone 220×4.5/( 5+6+9+2.5)×82226.37=3617960 yuan,

water 4.1×2.5/(5+6+9+2.5)×82226.37=37458 yuan,

shell 10×4.5/(5+6+9+2.5)×82226.37=164452 yuan,

The aggregate expenditure for concrete amounts to 35,449,613 yuan, equivalent to 4,878,303.68 US dollars. Similarly, the overall expenditure for concrete with a 30% shell replacement rate amounts to 36,896,717 yuan, equivalent to US$5,075,561.87. And the total cost of concrete with a 10% shell replacement rate is 38,343,981 yuan, approximately US$5,274,638.01.
