# Supplementary material for: Effect of biological shells aggregate on the mechanical properties and sustainability of concrete
Source: Sci Rep. 2024 May 9;14:10615. doi: 10.1038/s41598-024-61301-1 (PMC11078922; doi:10.1038/s41598-024-61301-1)
Supplement: Supplementary file 2 — Supplementary Information 2. [file 41598_2024_61301_MOESM2_ESM.docx]

# Appendix 2

The ratio of raw concrete, as calculated based on the quota, is m (cement) : m (sand) : m (stone) : m (water) = 5: 6: 9: 2.5:

cement 0635×5/(5+6+9+2.5)×3389.2×2400=1147890 yuan,

sand 180×6/(5+6+9+2.5)×3389.2=162681 yuan,

stone 220×9/(5+6+9+2.5) ×3389.2=298249 yuan,

water 4.1×2.5/(5+6+9+2.5)×3389.2=1543 yuan,

so the total cost of concrete is 1,610,363 yuan, or 220,582.56 in US dollars.

The ratio of 50% concrete aggregate is: m (cement) : m (sand): m (shell) : m (stone) : m (water) = 5: 6: 4.5: 4.5: 2.5, calculated according to the quota:

cement 0635×5/(5+6+9+2.5)×3389.2×2400=1147890 yuan,

sand 180×6/(5+6+9+2.5)×3389.2=162681 yuan,

stone 220×4.5/(5+6+9+2.5×3389.2=149124 yuan,

shell 10×4.5/(5+6+9+2.5)×3389.92=6779 yuan,

water 4.1×2.5/(5+6+9+2.5)×3389.2=1543 yuan,

Therefore, the total cost of concrete is 1,461,238 yuan, which is US$200,155.88. Similarly, the total cost of concrete with a 30% shell replacement rate is 1,520,888 yuan, or US$209,214.94, and the total cost of concrete with a 10% shell replacement rate is 1,580,538 yuan, or US$217,420.46 .
