# Supplementary material for: Effect of biological shells aggregate on the mechanical properties and sustainability of concrete
Source: Sci Rep. 2024 May 9;14:10615. doi: 10.1038/s41598-024-61301-1 (PMC11078922; doi:10.1038/s41598-024-61301-1)
Supplement: Supplementary file 3 — Supplementary Information 3. [file 41598_2024_61301_MOESM3_ESM.docx]

# Appendix 3

The original concrete mixture ratio was m (cement): m (sand): m (stone): m (water) = 5:6:9:2.5, as calculated based on the established quota for traditional aggregate:

cement 0635×5/(5+6+9+2.5)×263124.38×2400=89111456yuan,

sand180×6/(5+6+9+2.5)×263124.38=6393913 yuan,

stone 220×9/(5+6+9+2.5)×263124.38=23154912 yuan,

water 4.1×2.5/(5+6+9+2.5)×263124.38=119867 yuan,

so the total cost of concrete is 118,780,148 yuan, or 16,270,138.76 in US dollars.

The ratio of 50% concrete aggregate is: m (cement) : m (sand): m (shell) : m (stone) : m (water) = 5: 6: 4.5: 4.5: 2.5, calculated according to the quota:

cement 0635×5/(5+6+9+2.5)×263124.38×2400=89111456 yuan,

sand 180×6/(5+6+9+2.5)×263124.38=6393913 yuan,

stone 220×4.5/(5+6+9+2.5)×263124.38=11577456 yuan,

water 4.1×2.5/(5+6+9+2.5)×263124.38=119867 yuan,

shells is 10×4.5/(5+6+9+2.5)×263124.38=526248 yuan,

So the total cost of concrete is 107,202,692 yuan, or US$14,684,294.50. Similarly, the total cost of concrete with a 30% shell replacement rate is 111,833,674 yuan, or US$15,383,956.81, and the total cost of concrete with a 10% shell replacement rate is 116,464,656 yuan, or US$16,020,999.52.
