# Supplementary material for: Effect of biological shells aggregate on the mechanical properties and sustainability of concrete
Source: Sci Rep. 2024 May 9;14:10615. doi: 10.1038/s41598-024-61301-1 (PMC11078922; doi:10.1038/s41598-024-61301-1)
Supplement: Supplementary file 4 — Supplementary Information 4. [file 41598_2024_61301_MOESM4_ESM.docx]

# Appendix 4

Traditional aggregate concrete :

C1a = 0.0005× (40×1.195 + 96 × 2.618) + 0.0009 × (1.17× 1.195 + 0.723 ×3.178) + 0.0006 × (1.5× 1.195 + 0.8× 3.178）+0.00025×（0.29×1.195）=0.1556 kg

Shell replaces 10% concrete:

C1a = 0.0005× (40×1.195 + 96 ×2.618) + 0.00081× (1.17× 1.195 + 0.723×3.178) + 0.0006× (1.5× 1.195 + 0.8 ×3.178）+0.00025×（0.29×1.195）=0.1553 kg

Shell replaces 30% of the concrete:

C1a = 0.0005× (40×1.195 + 96 ×2.618) + 0.00063 × (1.17× 1.195 + 0.723×3.178) + 0.0006 × (1.5× 1.195 + 0.8 ×3.178）+0.00025×（0.29×1.195）=0.1546 kg

Shell replaces 50% of the concrete:

C1a = 0.0005× (40×1.195 + 96 ×2.618) + 0.00045 × (1.17× 1.195 + 0.723 ×3.178) + 0.0006× (1.5× 1.195 + 0.8 ×3.178）+0.00025×（0.29×1.195）=0.1539 kg
